# Exploring the role of traditional Chinese medicine in sarcopenia: mechanisms and therapeutic advances

**DOI:** 10.3389/fphar.2025.1541373

**Published:** 2025-06-30

**Authors:** Jianjun Yao, Sheng Xia

**Affiliations:** ^1^ Department of Geriatrics, Zhejiang Medical & Health Group Quzhou Hospital (Zhejiang Quhua Hospital), Quzhou, Zhejiang, China; ^2^ Department of Geriatrics, Wenzhou Geriatric Hospital, Wenzhou, Zhejiang, China

**Keywords:** sarcopenia, traditional Chinese medicine, muscular atrophy, natural drug, complementary and alternative therapies

## Abstract

Sarcopenia is characterized by the progressive loss of muscle mass, strength, and function, predominantly affecting the elderly population, and has emerged as a significant public health concern in the context of global aging. Its pathogenesis is multifactorial, involving aging-related processes, imbalances in skeletal muscle homeostasis, mitochondrial dysfunction, immune-mediated inflammation, and gut microbiota dysbiosis. Recent studies suggest that traditional therapies, including traditional Chinese medicine (TCM)—such as herbal medicine, acupuncture, and qigong—when integrated with modern medical treatments, may offer a more personalized therapeutic approach for older adults with sarcopenia. This integrative strategy has demonstrated considerable potential to improve muscle mass, enhance strength, decelerate the aging process, and ultimately improve patients’ quality of life. This review aims to summarize the clinical research and applications of TCM in sarcopenia management, explore the potential mechanisms underlying TCM’s therapeutic effects, and discuss future research directions and their clinical relevance.

## 1 Introduction

Sarcopenia, commonly referred to as muscle wasting, has seen continuous evolution in its definition and diagnostic criteria since the term was first introduced in the 1980s. Working Group on Sarcopenia in Older People (EWGSOP) proposed the first widely accepted diagnostic criteria, identifying low muscle mass, reduced muscle strength, and poor physical performance as the principal diagnostic indicators (Cruz-Jentoft et al., 2010). Recognizing the need for clarity and clinical relevance, EWGSOP revised its criteria in 2018, officially defining sarcopenia as a progressive and systemic skeletal muscle disorder characterized by accelerated loss of muscle mass and function. Importantly, the updated guidelines emphasized low muscle strength as the primary characteristic, with diagnosis confirmed by assessing muscle quantity and quality. Poor physical performance was further specified as an indicator of severe sarcopenia (Cruz-Jentoft and Sayer, 2019; Cruz-Jentoft et al., 2019).

In the Chinese population, where aging is rapidly accelerating, sarcopenia has attracted increasing concern. Epidemiological studies report that sarcopenia affects 5.7%–23.9% of individuals aged 60 and older, with higher prevalence observed in men and in eastern regions compared to western areas. The incidence also rises substantially with advancing age (Cui et al., 2023). Extensive research has confirmed that sarcopenia is strongly linked to frailty, increased risk of falls and fractures, functional impairment, cognitive decline, and elevated morbidity and mortality rates. Furthermore, with rising global life expectancy, sarcopenia has emerged as a critical public health issue, posing significant challenges to healthcare systems and profoundly affecting the quality of life of older adults (Beaudart et al., 2023; Zuo et al., 2023).

Despite advances in exercise therapy, nutritional supplementation, and pharmacological treatments, managing sarcopenia remains challenging, especially in elderly patients with multimorbidity and limited mobility. Traditional Chinese Medicine (TCM) has gained attention for its holistic approach, multi-target mechanisms, and favorable safety profile. TCM therapies—including herbal medicine, acupuncture, and traditional exercises like Tai Chi and Baduanjin—not only improve muscle mass and strength but also modulate inflammation, oxidative stress, mitochondrial function, and neuromuscular connectivity. This review summarizes recent advances in TCM research for sarcopenia and explores its mechanisms and therapeutic potential (The literature search strategy is detailed in the Supplementary Material).

## 2 Mechanisms of sarcopenia

Muscle mass and strength progressively decline with age, particularly in individuals over 75 years old. Studies indicate that the annual loss of muscle mass averages 0.64%–0.7% in women and 0.8%–0.98% in men, with losses typically more pronounced in the lower limbs than in the upper limbs. Notably, muscle function deteriorates even more rapidly: by age 75, muscle strength declines by approximately 3%–4% per year in men and 2.5%–3% in women (Amaral et al., 2014; Wilkinson et al., 2018a).

This reduction in strength is not solely attributable to the loss of lean muscle mass but also reflects a range of physiological and neural alterations, including diminished autonomic drive, impaired neuromuscular control (e.g., reduced motor neuron firing rates and slower nerve conduction velocity), increased non-contractile adipose infiltration, and reduced efficiency of excitation-contraction coupling (Gonzalez-Freire et al., 2014; Distefano and Goodpaster, 2018). Collectively, these factors accelerate the decline in muscle strength and function, adversely affecting mobility and quality of life in older adults.

**FIGURE 1:**
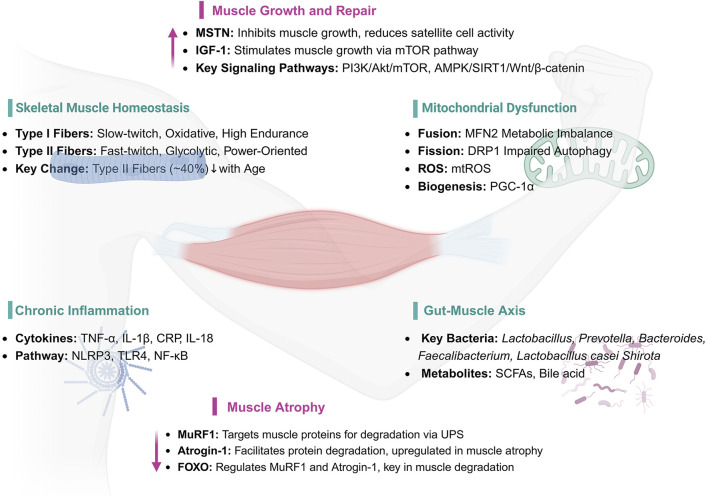
Key Mechanisms and Pathways in Muscle Health and Atrophy. IGF-1: Insulin-like Growth Factor 1, MSTN: Myostatin (Growth Differentiation Factor 8, GDF-8), AKT1: Protein Kinase B, PI3K: Phosphoinositide 3-Kinase, mTOR: Mechanistic Target of Rapamycin, AMPK: AMP-activated Protein Kinase, SIRT1: Sirtuin 1, PGC-1α: Peroxisome Proliferator-Activated Receptor Gamma Coactivator 1-alpha, MuRF1: Muscle Ring Finger 1, Atrogin-1: Atrophy-related ubiquitin ligase 1, FOXO: Forkhead Box O, MFN2: Mitofusin 2, DRP1: Dynamin-related Protein 1, ROS: Reactive Oxygen Species, mtROS: Mitochondrial Reactive Oxygen Species, TNF-α: Tumor Necrosis Factor-alpha, IL-1β: Interleukin-1 beta, CRP: C-reactive Protein, IL-18: Interleukin-18, NLRP3: NOD-like Receptor Pyrin Domain Containing 3, TLR4: Toll-like Receptor 4, NF-κB: Nuclear Factor-kappa B, UPS: Ubiquitin-Proteasome System, SCFAs: Short-chain Fatty Acids.

The pathogenesis of sarcopenia is a multifactorial and multi-pathway process, integrating diverse cellular and molecular mechanisms. Beyond the classical imbalance between protein synthesis and degradation, recent research highlights additional contributors such as disruption of skeletal muscle homeostasis, compromised mitochondrial quality control, dysregulated immune responses, and alterations in gut microbiota composition, all of which play critical roles in the onset and progression of sarcopenia (Cruz-Jentoft and Sayer, 2019; Picca and Calvani, 2021) (Figure 1).

### 2.1 Skeletal muscle homeostasis

The dynamic balance of skeletal muscle proteins is governed by the interplay between protein synthesis and degradation, with the net balance reflecting the difference between these two processes. This equilibrium is essential for preserving muscle mass and function (Schiaffino et al., 2013). Under normal physiological conditions, skeletal muscle homeostasis is maintained through intricate regulatory mechanisms that balance muscle hypertrophy and regeneration. However, aging progressively disrupts this balance, resulting in declines in both muscle mass and function. Muscle atrophy—a morphological adaptation—manifests as narrower muscle fibers, reductions in total muscle mass and protein content, diminished force production, and shifts in muscle fiber type composition (Tieland et al., 2018).

Skeletal muscle, the largest tissue in the human body, possesses robust regenerative capacity. It comprises bundles of type I (slow oxidative) and type II (fast oxidative/glycolytic) fibers and hosts a variety of mononuclear cells within its specialized microenvironment. Type I fibers primarily depend on oxidative metabolism, whereas type II fibers generate energy predominantly through glycolysis. With aging, the number of type II fibers declines by approximately 40%, contributing to substantial reductions in lean muscle mass. The preferential loss of type II fibers is a hallmark of sarcopenia and plays a central role in its pathogenesis (Deschenes, 2004; Phu et al., 2015).

### 2.2 Mitochondrial dysfunction

Mitochondrial dysfunction has been recognized as a central mechanism in skeletal muscle aging and sarcopenia.Mitochondria, double-membraned organelles present in most cells, serve as the primary site for cellular aerobic respiration and function as central hubs for metabolism and signal transduction. They play a critical role in maintaining mitochondrial dynamics—including fission, fusion, and autophagy—and are involved in processes such as ATP synthesis, fatty acid oxidation, calcium homeostasis, phospholipid synthesis, and the generation of reactive oxygen species (ROS) (Hood et al., 2019; Lin et al., 2022). When mitochondrial quality control mechanisms fail, structural and functional damage ensues, leading to muscle degeneration. In aging muscle cells specifically, significant changes in mitochondrial morphology and function are observed, accompanied by increased ROS levels, further exacerbating the aging process. Age-related impairments in mitochondrial fission and fusion diminish the cell’s capacity to clear damaged mitochondria, resulting in ROS-induced DNA damage and accelerated aging (Li J. et al., 2022).

During aging, reduced levels of the mitochondrial fusion protein mitofusin 2 (Mfn2) are closely associated with metabolic dysregulation and sarcopenia. Loss of Mfn2 triggers aging-related features, such as decreased mitophagy and impaired mitochondrial function, leading to skeletal muscle metabolic imbalance and muscle wasting. Research has shown that Mfn2 deficiency weakens autophagy processes, reduces mitochondrial quality, and further worsens age-related mitochondrial dysfunction and muscle atrophy (Sebastián et al., 2016). Additionally, muscle-specific deletion of the fission protein dynamin-related protein 1 (DRP1) results in significant muscle atrophy and weakness. Persistent DRP1 deficiency not only suppresses normal muscle growth but can also cause lethality, while inducible deficiency leads to muscle atrophy and degeneration. DRP1-deficient mice exhibit abnormal mitochondrial morphology, including increased size and functional impairment. Dysfunctional mitochondria further activate nuclear signaling pathways, inducing the ubiquitin-proteasome system and the unfolded protein response (Favaro et al., 2019; Dulac et al., 2021).

Mitochondrial biogenesis, referring to the formation of new mitochondria within cells, involves mitochondrial DNA replication, transcription, and the synthesis of associated proteins. This process is regulated by key transcription factors such as peroxisome proliferator-activated receptor gamma coactivator 1-alpha (PGC-1α) and is essential for maintaining cellular energy metabolism, antioxidant capacity, and stress-adaptive responses. During mitochondrial biogenesis, Migliavacca et al. conducted transcriptomic analyses on muscle biopsy samples from 119 elderly men and found that patients with sarcopenia commonly exhibited significant mitochondrial dysfunction. This dysfunction was characterized by downregulation of the PGC-1α/estrogen-related receptor alpha (ERRα) signaling pathway and reduced expression of genes involved in oxidative phosphorylation (Migliavacca et al., 2019). Further studies demonstrated that overexpression of PGC-1α activated mitochondrial metabolism and angiogenesis, significantly improving endurance, suggesting its potential role in preventing age-related sarcopenia (Yang et al., 2020). Subsequent experiments revealed that muscle-specific PGC-1α knockout mice displayed more severe sarcopenia compared to wild-type mice, with significantly reduced levels of Sestrin2 and phosphorylated ribosomal protein S6 kinase one (p-S6K1) in the white gastrocnemius muscle. Treatment with recombinant Sestrin2 improved muscle function and restored p-S6K1 levels in these mice (Fu et al., 2024).

Muscle stem cells, or satellite cells, play a crucial role in muscle regeneration following injury. Mitochondrial dysfunction not only damages mature muscle fibers but also impairs satellite cells, which are essential for muscle repair and regeneration. Age-related mitochondrial dysfunction leads to a decrease in satellite cell numbers and regenerative capacity, further accelerating the progression of sarcopenia. Disruption of mitochondrial homeostasis within satellite cells impairs their self-renewal and differentiation abilities, limiting muscle repair and the capacity to adapt to physical stress (Brooks et al., 2018; Greising et al., 2019; Marzetti et al., 2024).

Moreover, mitochondrial dysfunction interacts with chronic inflammation, creating a vicious cycle that accelerates muscle loss. Damaged mitochondria produce excessive ROS, which activate inflammatory pathways such as the nuclear factor kappa B (NF-κB), leading to upregulation of pro-inflammatory cytokines including interleukin-6 (IL-6) and tumor necrosis factor-alpha (TNF-α). These cytokines, in turn, further impair mitochondrial function, establishing a persistent feed-forward loop that promotes inflammation and muscle degeneration (Marzetti et al., 2013; Reid and Alexander, 2021).

### 2.3 Chronic inflammation

Aging is characterized by chronic, low-grade inflammation. As individuals age, the accumulation of inflammatory factors promotes muscle catabolism, contributing to the loss of muscle mass and strength and exacerbating age-related conditions such as sarcopenia (Jimenez-Gutierrez et al., 2022). A meta-analysis covering 17 studies and involving 11,249 participants (3,072 with sarcopenia and 8,177 without) showed that C-reactive protein (CRP) levels were significantly higher in sarcopenia patients compared to the control group, suggesting an association between sarcopenia and elevated CRP levels (Bano et al., 2017). 2019 clinical study conducted in Beijing, it was found that serum inflammatory markers, such as TNF-α, TNF-like weak inducer of apoptosis (TWEAK), IL-18, were significantly elevated in sarcopenia patients, while insulin-like growth factor 1 (IGF-1) and adiponectin, which are associated with metabolism, were significantly lower. Furthermore, enhanced lifestyle interventions significantly improved muscle mass and reduced serum levels of related inflammatory factors in sarcopenia patients (Li C. et al., 2019).

At the molecular level, inflammatory factors contribute to pyroptosis—a form of programmed cell death—in muscle cells, accelerating catabolism and further reducing muscle mass and strength. The NLRP3 (NOD-like receptor family pyrin domain containing 3) inflammasome plays a pivotal role in this process by promoting the maturation and secretion of pro-inflammatory cytokines such as IL-1β and IL-18. This activation enhances both local and systemic pyroptotic and inflammatory responses, damages mitochondrial function, and reduces glycolytic capacity in muscle cells, all of which contribute to muscle wasting and impaired fiber growth (Busch et al., 2021; Ma, 2023).

Inflammatory stimuli also activate NLRP3 to induce acute mitochondrial reactive oxygen species (mtROS) production, triggering oxidative stress that damages muscle cells, inhibits growth, and accelerates atrophy (Antuña et al., 2024; Yungi et al., 2024). Additionally, the NF-κB signaling pathway plays a critical role in sarcopenia pathogenesis. NF-κB overactivation inhibits the proliferation and differentiation of muscle satellite cells, reducing muscle regenerative capacity, while also promoting oxidative stress through increased mtROS production, further impairing muscle cell function and growth (Sun et al., 2018; Lee et al., 2024).Furthermore, Alexander et al. found that Toll-like receptor 4 (TLR4) promotes muscle protein degradation by coordinating the activation of the ubiquitin-proteasome pathway and the autophagy-lysosome pathway, leading to muscle atrophy. This finding provides a potential intervention target for inflammation-related muscle atrophy treatment (Doyle et al., 2011).

### 2.4 Gut-muscle axis

The human gastrointestinal tract harbors a vast microbiome, containing approximately 100 trillion microorganisms, including bacteria, archaea, and fungi (Eckburg et al., 2005). These microorganisms are crucial for human health and many functional characteristics of the gut microbiome. They assist in the digestion of dietary polysaccharides, metabolize xenobiotic drugs, promote immune responses, and prevent pathogen invasion (Zmora et al., 2019; Erttmann et al., 2022). Dysbiosis of the gut microbiome is closely associated with the decline in muscle mass and physical function, particularly in the elderly population. As individuals age, the composition of the gut microbiome undergoes changes, which may be closely linked to sarcopenia and the decline in physical function during aging. Increasing research has begun to explore the interaction between the gut microbiome and muscle health, revealing the potential mechanisms behind the so-called “gut-muscle axis” (Liu et al., 2021; Zhang T. et al., 2022).

Active adults typically have higher muscle mass and better physical function than sedentary adults, and their gut microbiomes also exhibit different dominant bacterial populations (Bressa et al., 2017). One study showed that in the stool samples of elderly individuals with higher frailty scores, the number of *Lactobacillus* was significantly reduced, with a decrease of up to 26-fold. Additionally, populations of *Bacteroides*/*Prevotella* and *Firmicutes* were also significantly reduced, by 3-fold and 4-fold, respectively (van Tongeren et al., 2005). Moreover, some studies have also explored the effects of probiotic supplements on lean body mass and physical performance, finding that supplementation with probiotics improved muscle strength, athletic performance, and recovery in athletes (Marttinen et al., 2020; Przewłócka et al., 2020).

The gut microbiome and its metabolites play a critical role in maintaining muscle health and function. Specifically, bile acids and short-chain fatty acids (SCFAs) are considered important mediators through which gut microbiota influence muscle health. Dysbiosis of the gut microbiota can lead to a range of negative effects, one of which is excessive muscle atrophy (Mancin et al., 2023). Germ-free mice, lacking a healthy gut microbiota, exhibited significant skeletal muscle atrophy, closely associated with the upregulation of muscle atrophy-related genes such as Forkhead box O3 (FoxO3), Atrogin-1, and Muscle Ring Finger-1 (MuRF-1) in their skeletal muscle. At the same time, genes related to muscle generation and functional recovery, particularly myosin heavy chain genes, were significantly downregulated in these mice (Lee et al., 2023; Kang et al., 2024). Lahiri et al. further revealed that the muscle atrophy in germ-free mice was closely related to a reduction in IGF-1 levels and a decline in the transcription of genes associated with skeletal muscle growth and mitochondrial function. Encouragingly, when the gut microbiota from pathogen-free mice was transplanted into germ-free mice, the skeletal muscle mass of the germ-free mice was significantly restored, muscle atrophy markers decreased, and the expression of genes related to neuromuscular junction formation, such as Rapsyn and Lrp4, was significantly increased. Additionally, SCFA treatment also effectively improved the skeletal muscle health of germ-free mice, partially reversing muscle damage induced by gut microbiota dysbiosis (Lahiri et al., 2019). Recent research further confirms the impact of the gut microbiome on age-related muscle degeneration. Researchers found that treatment with *Lactobacillus casei Shirota* for 12 weeks in SAMP8 mice (Senescence-Accelerated Mouse Prone 8) significantly reduced age-related muscle mass and strength decline. This study also suggested that *L. casei Shirota* treatment might slow muscle degeneration during aging by restoring SCFA levels, modulating inflammation, improving mitochondrial function, and optimizing the diversity of the gut microbiome (Chen et al., 2022).

### 2.5 Cancer-associated sarcopenia

Cancer-associated sarcopenia refers to the progressive decline in skeletal muscle mass, strength, and function observed in patients with malignancies. Unlike primary sarcopenia associated with aging, cancer-associated sarcopenia tends to develop earlier, progress more rapidly, and is frequently accompanied by cancer cachexia. Epidemiological studies indicate that approximately 50%–80% of patients with advanced cancer experience varying degrees of muscle wasting (Zhang et al., 2023). There exists a complex and bidirectional relationship between sarcopenia and cancer. On one hand, cancer induces sarcopenia through mechanisms such as systemic inflammation, metabolic dysregulation, elevated catabolic cytokines (e.g., IL-6, TNF-α), and the direct myotoxic effects of chemotherapy and radiotherapy. Cancer cachexia represents the extreme manifestation of this process, characterized by severe weight loss, muscle atrophy, and elevated systemic inflammation. On the other hand, sarcopenia itself acts as an independent adverse prognostic factor. Patients with cancer-related sarcopenia exhibit reduced chemotherapy tolerance, increased incidence of treatment-related toxicities, diminished physical performance, prolonged hospitalization, and significantly higher mortality rates (Williams et al., 2021; Fang et al., 2023; Kiss et al., 2023).Additionally, sarcopenia limits patients’ eligibility for surgical or targeted therapies, further compromising treatment outcomes (Levolger et al., 2015). Breaking this vicious cycle between cancer and sarcopenia is therefore critical for improving clinical prognosis.

At the mechanistic level, cancer-associated sarcopenia is driven by a series of interconnected pathological processes (Moreira-Pais et al., 2021). Systemic inflammation leads to sustained elevation of pro-inflammatory cytokines, which activate key catabolic signaling pathways such as NF-κB and Janus kinase/signal transducer and activator of transcription 3 (JAK/STAT3) axes. These pathways upregulate the expression of muscle-specific E3 ubiquitin ligases, including muscle RING-finger protein-1 (MuRF1) and Atrogin-1, accelerating skeletal muscle proteolysis through the ubiquitin-proteasome system (Martin et al., 2023). Metabolic disorders, including insulin resistance and elevated basal energy expenditure, combined with mitochondrial dysfunction, further impair muscle energy homeostasis (Gicquel et al., 2024).

Recently, accumulating evidence has indicated that TCM may provide a promising complementary approach for alleviating cancer-associated sarcopenia (Li S. et al., 2025). Astragalus membranaceus extracts, particularly astragaloside IV, have demonstrated the ability to suppress oxidative stress and inflammation, modulate the activity of the ubiquitin-proteasome system and autophagy-lysosomal pathways, and consequently reduce skeletal muscle proteolysis (Liu et al., 2025). In addition, Polygonatum sibiricum polysaccharides have been reported to activate the Phosphatidylinositol 3-kinase/Protein kinase B/Mammalian target of rapamycin (PI3K/Akt/mTOR) signaling pathway, thereby improving mitochondrial dysfunction, enhancing muscle energy metabolism, and promoting muscle cell homeostasis (Li Y. et al., 2025).

## 3 Clinical research on traditional Chinese medicine and sarcopenia

In TCM, sarcopenia is not explicitly discussed as a standalone condition, but its clinical manifestations are often integrated into the broader categories of “Wei syndrome,” “deficiency syndrome,” “bi syndrome of flesh,” and “bi syndrome of bone.” The clinical symptoms of Wei syndrome were first described in the Huangdi Neijing (Yellow Emperor’s Inner Classic), where it is noted: “Wei means weakness, and weakness is caused by movement.” Wei syndrome primarily affects the muscles and bones of the limbs, with the lesions of the five viscera (zangfu organs) potentially leading to Wei syndrome. Wei Lun (in the “Plain Questions” of the Huangdi Neijing) emphasized that “in the Yangming, the sea of the five zangfu and six fu is primarily responsible for running the zongjin, and the zongjin is responsible for binding the bones, but also for strengthening the organs.” It also presents the therapeutic principle of “treating erectile dysfunction by regulating the Yangming,” underscoring the importance of the spleen and stomach in addressing dysfunctions of other zangfu organs.

**FIGURE 2:**
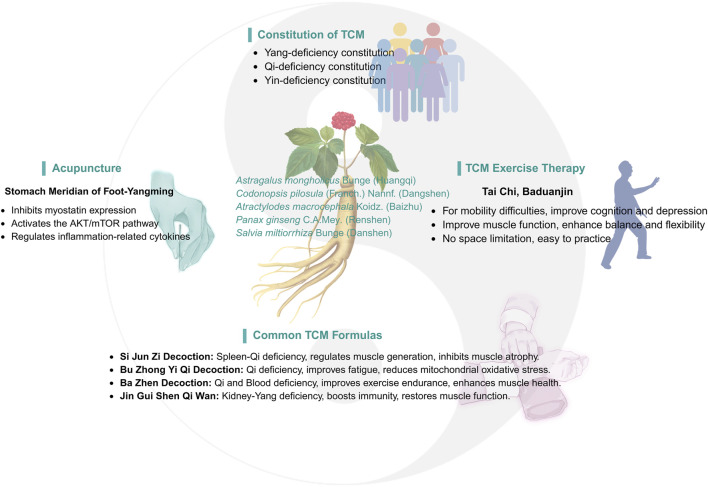
Clinical research on traditional Chinese medicine and sarcopenia.

As part of a comprehensive approach, TCM has demonstrated significant efficacy and unique advantages in treating sarcopenia. TCM emphasizes a holistic approach and syndrome differentiation, aiming to restore balance in the body by regulating Yin and Yang, qi and blood, and the zangfu organs. This balance helps enhance the body’s self-healing ability. TCM treatments for sarcopenia are varied and flexible, including Chinese herbal medicine (CHM), acupuncture, massage, qigong, and moxibustion. These therapies not only improve muscle mass and function but also promote overall health, particularly among the elderly (Figure 2).

### 3.1 Traditional Chinese medicine constitution

In TCM theory, the classification of constitution types provides a personalized treatment framework for clinical treatment. Professor Wang Qi, a great physician of Chinese medicine, proposed nine constitutions classification (Li L. et al., 2019),including one balanced constitution (neutral constitution) and eight biased constitutions (Qi deficiency, Yang deficiency, Yin deficiency, blood stasis, Qi stagnation, dampness and heat, phlegm dampness and special constitution), which provides a new perspective for the diagnosis and treatment of senile diseases such as sarcopenia.

With aging, elderly individuals experience a gradual decline in nutritional intake and immune function, along with reduced ability to eat, digest, and absorb nutrients. This can lead to muscle strength loss, decreased energy, and reduced internal reserves, contributing to the development of various biased constitutions. Research has shown that constitutional types such as Qi deficiency, Yang deficiency, Yin deficiency, and blood stasis are closely associated with sarcopenia, particularly in elderly males. Qi deficiency and Yang deficiency are considered potential risk factors for sarcopenia, providing targets for prevention and intervention in clinical treatment (Shi et al., 2018; Nie et al., 2024). Another study indicated that Yin deficiency was independently correlated with sarcopenia, and may serve as a potential risk factor for the condition (Wang C. et al., 2024).

Based on the varying physical manifestations, TCM offers tailored treatment programs. For example, for patients with yang deficiency, commonly used herbs to warm and tonify kidney yang include *Zingiber officinale* Roscoe (Ganjiang), *Cinnamomum cassia* (L.) D. Don (Rougui), *Aconitum carmichaelii* Debeaux (Fuzi), and others to warm yang and dispel cold. For those with qi deficiency, herbs such as *Astragalus mongholicus* Bunge (Huangqi), *Atractylodes macrocephala* Koidz. (Baizhu), *Panax ginseng* C.A.Mey. (Renshen) and *Codonopsis pilosula* (Franch.) Nannf. (Dangshen) are used to tonify qi and replenish vitality. For individuals with a blood stasis constitution, blood-activating herbs such as *Salvia miltiorrhiza* Bunge (Danshen), *Kalanchoe pinnata* (Lam.) Pers. (Sanqi), and Carthamus tinctorius *L.* (Honghua) are commonly used to promote blood circulation and alleviate blood stasis symptoms (Table 1).

**TABLE 1:** Body constitution/patterns associated with sarcopenia, recommended herbs, and Representative Prescriptions.

Syndrome/Constitution	Main manifestations	Recommended herbs	Representative formula
Qi Deficiency	Fatigue, poor appetite, shortness of breath, spontaneous sweating	*Astragalus mongholicus* Bunge (Huangqi), *Codonopsis pilosula* (Franch.) Nannf. (Dangshen)*, Atractylodes macrocephala* Koidz. (Baizhu)*, Panax ginseng* C.A.Mey. (Renshen)	Sijunzi Decoction
Blood Deficiency	Palpitations, poor memory, pale complexion, dizziness	*Angelica sinensis (*Oliv.) Diels (Danggui)*, Rehmannia glutinosa* (Gaertn.) Libosch. ex DC. (Shudi)*, Ziziphus jujuba* Mill. (Dazao)*, Spatholobus suberectus* Dunn (Jixueteng)	Siwu Decoction
Yang Deficiency	Soreness and weakness of the lower back and knees, cold intolerance, fatigue	*Cinnamomum cassia* (L.) D. Don (Rougui)*, Taxillus sutchuenensis *(Lecomte) Danser (Sangjisheng*), Aconitum carmichaelii* Debeaux (Fuzi)*, Zingiber officinale* Roscoe (Ganjiang)	Jingui Shenqi Wan
Yin Deficiency	Dry mouth and throat, tidal fever, night sweats, restlessness	*Asparagus cochinchinensis* (Lour.)Merr. (Tiandong)*, Ophiopogon japonicus* (L. f.) Ker Gawl. (Maidong)*, Adenophora stricta* Miq. (Shashen)*, Scrophularia ningpoensis* Hemsl. (Xuanshen)	Shengmai Yin
Blood Stasis	Dark purplish lips and tongue, stabbing pain in limbs, symptoms worsening at night	*Salvia miltiorrhiza* Bunge (Danshen)*, Ligusticum sinense ‘Chuanxiong’* (Chuanxiong)*, Kalanchoe pinnata* (Lam.) Pers. (Sanqi)*, Carthamus tinctorius* L. (Honghua)	Taohong Siwu Decoction
Phlegm-Dampness	Obesity, excessive phlegm, heaviness in the limbs	*Pinellia ternata* (Thunb.) Ten. ex Breitenb. (Banxia)*, Citrus reticulata Blanco* (Chenpi)*, Atractylodes Lancea* (Thunb.) DC. (Cangzhu)*, Trichosanthes kirilowii* Maxim. (Gualou)	Erchen Decoction

### 3.2 Chinese herbal medicine

Chinese herbal medicine (CHM), as a core component of TCM, has attracted increasing attention and recognition in recent years. Since the discovery of artemisinin by Tu Youyou, the efficacy of CHM in treating various diseases has been supported by numerous high-quality studies (Tu, 2016; Chen W.-J. et al., 2023; Yang Y. et al., 2023).

CHM not only treats diseases that Western medicine has not yet effectively addressed but also demonstrates unique therapeutic advantages in many fields. In recent years, more studies have shown that CHM has a significant effect on sarcopenia as a complementary treatment. A systematic review and meta-analysis of 17 randomized controlled trials involving 1,440 participants demonstrated that CHM significantly improved multiple clinical outcomes in sarcopenic patients, including total effective rate (RR = 1.29, 95% CI: 1.21–1.36), muscle mass (SMD = 1.02, 95% CI: 0.55–1.50), grip strength (SMD = 0.66, 95% CI: 0.36–0.96), 6-m walking speed (SMD = 1.34, 95% CI: 0.60–2.08), and scores on the Short Physical Performance Battery (SPPB) (mean increase: 1.50 points, 95% CI: 1.05–1.95) (Zhang et al., 2024a). These findings suggest that CHM, either alone or combined with conventional treatments, holds promise in improving muscle strength, mobility, and physical performance among elderly patients with sarcopenia.

From TCM perspective, sarcopenia is often associated with spleen-qi deficiency and kidney-yang deficiency. Commonly prescribed formulas include Sijunzi Decoction, Buzhong Yiqi Decoction, Bazhen Decoction, and Jingui Shenqi Wan. Clinical studies have shown that combining these herbal therapies with conventional treatments can significantly alleviate sarcopenia symptoms, particularly in elderly populations (Zhang et al., 2024b) (Table 2).

**TABLE 2:** Summary of clinical studies on traditional Chinese medicine for sarcopenia.

No.	References	Population	Intervention	Sample size (n)	Clinical outcomes	Duration
1	Wang et al. (2022b)	Elderly sarcopenia	Modified Sijunzi Decoction combined with resistance band training	100	Improved grip strength, ASMI, SPPB score, and nutritional status	8 weeks
2	Chen and Wen (2021)	Elderly sarcopenia	Buzhong Yiqi Decoction	40	Reduced serum IL-6 and TNF-α levels	8 weeks
3	Lv and Wang (2021)	Elderly sarcopenia	Buzhong Yiqi Decoction	80	Improved skeletal muscle mass, muscle strength, and muscle function	12 weeks
4	Chen (2022)	Elderly sarcopenia	Buzhong Yiqi Decoction combined with Baduanjin	60	Improved skeletal muscle mass and muscle strength, and reduced fall risk	22 weeks
5	Zhou (2024)	Elderly sarcopenia	Bazhen Decoction combined with nutritional support and exercise	118	Improved TCM syndrome score, 6-min walk distance, and skeletal muscle health	12 weeks
6	Hao and Hu (2021b)	Elderly chronic heart failure with sarcopenia	Bazhen Decoction	98	Improved cardiac function, skeletal muscle mass, and muscle strength	12 weeks
7	Jiang et al. (2024)	Elderly type 2 diabetes mellitus with sarcopenia	Shenling Baizhu San combined with exercise	140	Reduced HbA1c, improved grip strength and 6-m walking speed, and reduced fall risk	12 weeks
8	Huang et al. (2023a)	Elderly sarcopenia	Tai Chi	60	Improved lower limb neuromuscular response and dynamic balance	12 weeks
9	Zhu et al. (2019)	Elderly sarcopenia	Tai Chi	90	Improved muscle strength and balance	8 weeks
10	Peng et al. (2024)	Elderly sarcopenia	Baduanjin combined with blood flow restriction training	126	Improved SMI, ASMI, grip strength, SPPB score, and MBI	12 weeks
11	Xu et al. (2022)	Elderly sarcopenia	Baduanjin combined with resistance exercise	60	Improved grip strength, five-time sit-to-stand time, walking speed, and MBI	12 weeks

Abbreviations: ASMI, appendicular skeletal muscle mass index; SPPB, short physical performance battery; SMI, skeletal muscle mass index; MBI, modified barthel index; IL-6, Interleukin-6; TNF-α, Tumor Necrosis Factor-α; HbA1c, Glycated Hemoglobin.

Note: All interventions were performed in addition to standard care.

Sijunzi Decoction is composed of *P. ginseng* C.A.Mey*.* (Renshen), *A. macrocephala* Koidz. (Baizhu), *Poria* (Fulin) and *Glycyrrhiza uralensis* Fisch. (Gancao), which is mainly used for the treatment of spleen-stomach qi deficiency syndrome. The principle of treatment is mainly to benefit qi and strengthen the spleen, which can improve fatigue, regulate muscle production, inhibit muscle atrophy, and improve mitochondrial function (Chen J.-M. et al., 2019). Growing evidence suggests that integrating herbal therapy with physical rehabilitation may provide superior outcomes in managing sarcopenia. A randomized controlled trial involving 100 elderly patients demonstrated that an 8-week intervention with modified Sijunzi Decoction combined with resistance band training significantly improved clinical parameters compared to conventional therapy alone, including increased grip strength (23.75 ± 3.86 kg vs 20.05 ± 3.69 kg), appendicular skeletal muscle mass index (ASMI) (6.53 ± 0.41 kg/m^2^ vs 6.26 ± 0.32 kg/m^2^), and SPPB scores (9.54 ± 1.85 vs 8.14 ± 1.96), with all differences reaching statistical significance (Wang Q. et al., 2022).

Buzhong Yiqi Decoction is traditionally used to treat symptoms of qi deficiency, such as fatigue, tiredness, and loss of appetite. Its therapeutic effects are attributed to the regulation of the antioxidant system, thereby reducing mitochondrial oxidative stress damage, alleviating muscle weakness, and enhancing both physical health and cognitive function (Ding et al., 2024; Dong et al., 2024). A randomized controlled study demonstrated that modified Buzhong Yiqi Decoction combined with conventional intervention significantly reduced serum levels of IL-6 and TNF-α in elderly patients with sarcopenia after 2 months of treatment compared to conventional intervention alone (*P* < 0.05). Importantly, no significant changes were observed in white blood cell count, alanine aminotransferase, or serum creatinine, indicating that the combination therapy maintained a good safety profile (Chen and Wen, 2021).

Bazhen Decoction is composed of eight Chinese herbs, such as *P. ginseng* C.A.Mey. (Renshen), *A. macrocephala* Koidz. (Baizhu), *Rehmannia glutinosa* (Gaertn.) Libosch. ex DC. (Shudi), *Radix Angelicae sinensist* (Dangui), *Paeonia lactiflora* Pall. (Baishao). It has the functions of tonifying qi, nourishing blood, invigorating spleen and nourishing qi. This recipe is widely used to treat a variety of symptoms caused by qi and blood deficiency, especially in the elderly group, often used to intervene in sub-health status and prevent age-related degenerative diseases (Li C. et al., 2024). Recent clinical evidence supports its efficacy in sarcopenia management. A randomized controlled trial involving 118 elderly patients demonstrated that a 12-week intervention with Bazhen Decoction combined with nutrition and exercise significantly improved clinical outcomes compared to conventional therapy alone. Specifically, the combined treatment reduced clinical symptom scores (from 13.70 ± 2.68 to 3.89 ± 1.01 vs 13.56 ± 2.77 to 6.69 ± 1.30), increased 6-min walking distance (570.04 ± 35.01 m vs 533.08 ± 33.67 m), enhanced muscle function (1.01 ± 0.13 m/s vs 0.91 ± 0.11 m/s), improved muscle strength (23.38 ± 2.40 kg vs 22.49 ± 2.14 kg), and elevated muscle mass (5.87 ± 0.85 kg/m^2^ vs 5.40 ± 0.83 kg/m^2^), indicating superior clinical effectiveness (Zhou, 2024).

CHM has also shown good efficacy in the treatment of sarcopenia combined with other diseases. For example, patients with heart failure and sarcopenia treated with trimetazidine combined with Bazhen decoction can not only improve cardiac function, but also improve muscle mass and strength (Hao and Hu, 2021a). Similarly, recent clinical evidence from a randomized controlled trial involving 70 elderly patients with type 2 diabetes mellitus and sarcopenia showed that a 12-week intervention with Shenling Baizhu San combined with exercise achieved superior outcomes compared to conventional therapy alone. The combined treatment significantly lowered HbA1c levels (7.66% ± 0.58% vs 7.90% ± 0.70%), enhanced grip strength (24.00 ± 4.68 kg vs 22.24 ± 4.76 kg), and increased 6-m walking speed (1.06 ± 0.15 m/s vs 0.99 ± 0.16 m/s), although no significant difference was observed in skeletal muscle mass index (SMI) (Jiang et al., 2024). These findings suggest that CHM, when integrated with conventional therapies, may offer additional benefits in improving metabolic control, muscle strength, and physical performance in sarcopenic patients with comorbidities.

### 3.3 Traditional Chinese exercise

#### 3.3.1 Tai Chi

Exercise therapy, particularly resistance and aerobic training, is a primary approach for treating sarcopenia in the elderly (Bårdstu et al., 2020; Kakehi et al., 2022). Traditional Chinese exercises, originating from TCM with a history of over 3,000 years, integrate therapeutic, aerobic, and mind-body elements. Representative practices such as Tai Chi, Baduanjin, Wuqinxi, and Yijinjing are characterized by gentle movements and emphasis on relaxation. Studies have shown that these exercises promote muscle hypertrophy, enhance strength and endurance, prevent muscle atrophy, and improve physiological functions related to frailty, including blood pressure regulation and vascular function (Kasim et al., 2020; Niu et al., 2022; Wang L. et al., 2022).

Among these traditional sports, Tai Chi, as a combination of Tuina and boxing, integrates aerobic training, resistance training and flexibility training, and is considered to significantly promote the overall health of the elderly (Siu et al., 2021). A systematic review of 11 randomized controlled trials involving 1,676 older adults with sarcopenia or frailty found that Tai Chi significantly improved lower-limb function, including the 30-s chair stand test (WMD = 2.36, 95% CI: 1.50–3.21) and the timed up-and-go test (WMD = −0.72, 95% CI: −1.10 to −0.34), and reduced fall incidence (WMD = −0.41, 95% CI: −0.64 to −0.17) (Huang et al., 2022).

The elderly are often accompanied by mental health problems such as anxiety and depression. Tai Chi not only helps to enhance physical strength and flexibility, but also plays an important role in improving mental health, especially in the antidepressant effect. Studies have shown that Tai chi helps relieve depressive symptoms by regulating the autonomic nervous system, improving mood-related neural networks, and its anti-inflammatory effects. In addition, Tai chi can also reduce the susceptibility to dopaminergic neurodegeneration, thereby helping the elderly maintain cognitive function and emotional stability, and reduce the occurrence and development of depressive symptoms (Li G. et al., 2022).

#### 3.3.2 Baduanjin

It may be difficult for most older adults to adhere to the high-load resistance training programs recommended by the American College of Sports Medicine and the American Heart Association. Moreover, conventional exercise rehabilitation is often time-consuming, costly, and complex. Therefore, it is particularly important to identify safe and feasible exercise programs for elderly patients with sarcopenia.

Baduanjin, an ancient Chinese qigong practice originating in the Song Dynasty, involves gentle, rhythmic movements and has been shown to effectively improve the overall physical condition of the elderly. Regular practice of Baduanjin enhances balance, gait, and flexibility while also alleviating common psychological issues such as anxiety and depression. For elderly patients with sarcopenia, Baduanjin not only improves muscle function but also enhances overall quality of life, making it a highly suitable exercise modality for this population (Luo et al., 2023; Yang W. et al., 2023).

Compared with Tai Chi, Baduanjin is relatively static, involves a lower exercise load, and is easier to learn. It is not restricted by venue and can be practiced in any suitable space, which is particularly beneficial for older adults with mobility impairments. Studies have demonstrated that incorporating Baduanjin training into conventional rehabilitation programs significantly improves muscle mass, enhances muscle function, and promotes better daily activity performance and quality of life among the elderly (Xu et al., 2022; Zhou, 2024).

In a randomized trial with 126 older sarcopenic patients, Baduanjin combined with blood flow restriction training over 12 weeks led to notable improvements: handgrip strength rose by approximately 7 kg, Barthel Index improved by nearly 15 points, and SPPB scores increased by about two points. ASMI also showed significant gains. All these changes were markedly greater compared to the control group (*P* < 0.05), highlighting the effectiveness of the combined intervention in enhancing muscle strength, physical performance, and functional independence (Peng et al., 2024).

### 3.4 Acupuncture

Acupuncture, a core component of TCM with a history of over 3,000 years, has become one of the most widely recognized forms of alternative medicine worldwide. Techniques such as manual acupuncture, electroacupuncture, and transcutaneous electrical acupoint stimulation have been shown to exert therapeutic effects by stimulating specific acupoints, particularly in diseases that are challenging for modern medicine to treat (Liu et al., 2017; Wang Y. et al., 2023).

In clinical studies, the effects of acupuncture on sarcopenia have shown some variability. In a randomized controlled trial involving 60 patients with sarcopenia, twice-weekly electroacupuncture at yangming meridian acupoints—including bilateral Binao (LI14), Quchi (LI11), Zusanli (ST36), and Yanglingquan (GB34)—over 12 weeks significantly improved ASMI, grip strength, and body moisture percentage, while reducing 6-m walking time and body fat percentage compared with nutritional therapy alone (Ma et al., 2023). Similarly, another randomized controlled trial involving 42 elderly patients with sarcopenia (21 per group) reported that electroacupuncture combined with rehabilitation exercise training for 4 weeks significantly increased SPPB scores, 6-min walking distance, grip strength, and relative ASMI, and decreased SARC-F scores compared to exercise training alone (Ling and Huang, 2022).

However, contrasting results were observed in a study conducted in Brazil. In this trial, sarcopenic older adults (n = 11) received acupuncture treatment (24 sessions over 8 weeks), but no significant improvements were detected in lean mass (38.9 ± 6.7 kg, *P* = 0.348), body fat percentage (33.4% ± 6.1%, *P* = 0.358), handgrip strength (left hand at Post 3: 25.40 ± 8.54 kg), or functional performance as assessed by the Timed Up and Go (TUG) test (*P* = 0.86). Although IL-6 levels showed a slight reduction at Post 2 (2.31 ± 2.26 pg/mL, *P* = 0.05), TNF-α and IL-10 levels did not change significantly. Despite the lack of objective improvements, most participants reported subjective benefits, including pain relief and enhanced mobility (Soares Mendes Damasceno et al., 2019).

The mechanisms by which acupuncture treats sarcopenia involve multiple biological pathways, with electroacupuncture playing a key role in regulating skeletal muscle repair and regeneration. First, electroacupuncture inhibits the expression of myostatin, a negative regulator of muscle growth, and promotes satellite cell proliferation, thereby accelerating skeletal muscle repair and regeneration (Takaoka et al., 2007). Additionally, it suppresses the overactivation of the cyclic GMP-AMP synthase (cGAS)-stimulator of interferon genes (STING)-NF-κB signaling pathway, effectively reducing exercise-induced muscle inflammation and protecting skeletal muscle from damage (Li H. et al., 2024). Further studies have demonstrated that electroacupuncture can inhibit D-galactose-induced skeletal muscle atrophy, delay mitochondrial dysfunction associated with aging, and reduce muscle nuclear apoptosis (Jin et al., 2023).

Moreover, electroacupuncture significantly improves physical performance, muscle mass, and the cross-sectional area of the gastrocnemius muscle in SAMP8 mice. It promotes angiogenesis by activating AKT/mTOR/p70S6 kinase signaling pathway, upregulating hypoxia-inducible factor 1-alpha (HIF-1α) and vascular endothelial growth factor A (VEGF-A), and inhibiting the expression of muscle atrophy-related genes such as muscle ring finger protein-1 (MuRF1) and muscle atrophy F-box (MAFbx) (Zhu et al., 2020). In addition, in a rat model of spinal cord injury, electroacupuncture has demonstrated the potential to prevent neuromuscular junction degeneration, reduce muscle atrophy, improve neural function, and promote axonal regeneration (Zhang X. et al., 2022).

## 4 Basic research on sarcopenia of traditional Chinese medicine

### 4.1 Muscle synthesis and degradation pathways

**FIGURE 3:**
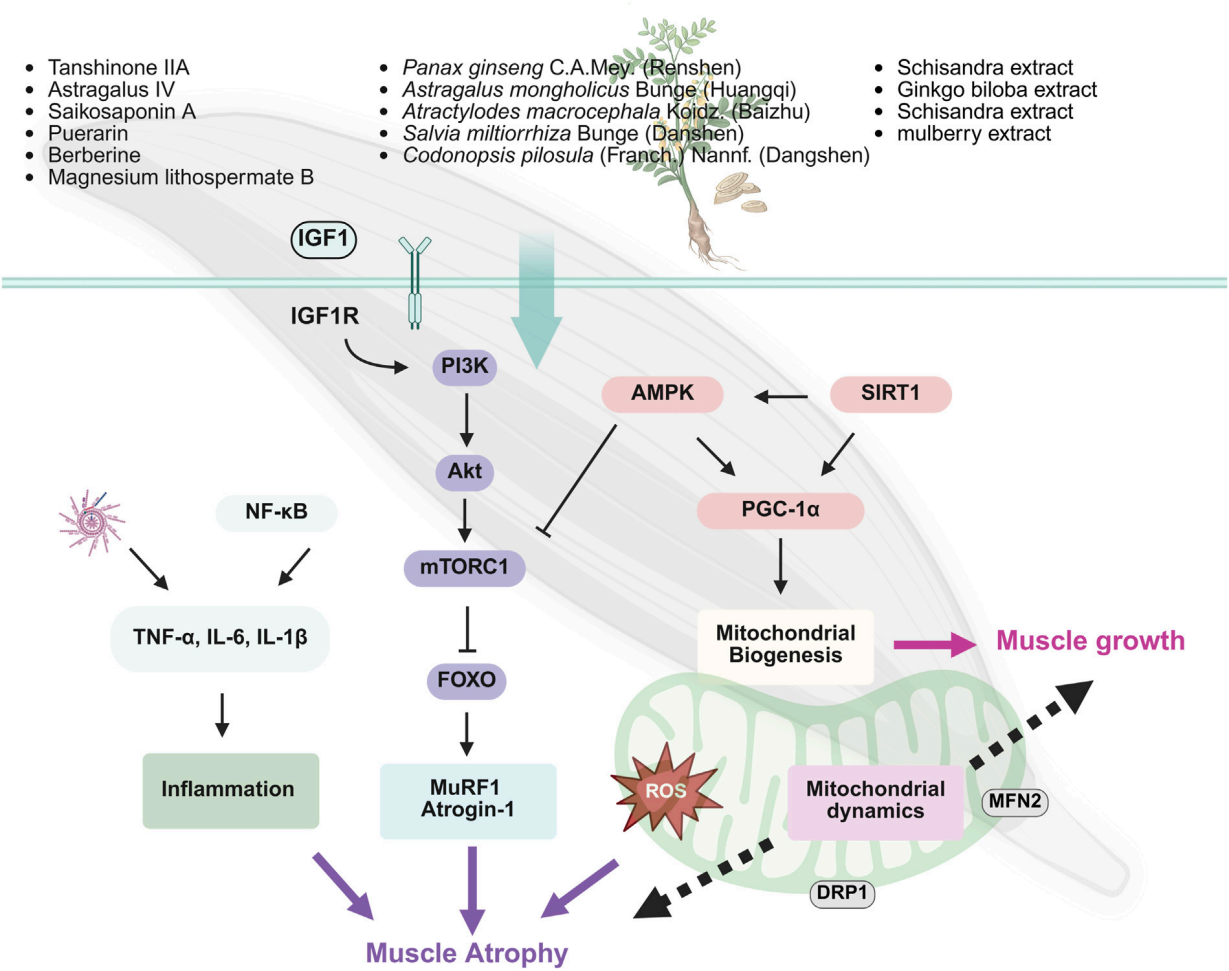
Basic research on sarcopenia of traditional Chinese medicine.

The occurrence of sarcopenia is closely related to the dysfunction of multiple signaling pathways regulating protein synthesis, particularly the imbalance between protein synthesis and degradation (Wilkinson et al., 2018b). Key mechanisms include the inhibition of PI3K/Akt/mTOR pathway and the overactivation of the adenosine 5′-monophosphate-activated protein kinase (AMPK) pathway. The inhibition of the PI3K/Akt/mTOR pathway reduces protein synthesis, while the activation of AMPK further suppresses mTOR activity, limiting muscle growth and repair and promoting muscle atrophy (Gellhaus et al., 2023; Kerr et al., 2024) (Figure 3).

TCM exhibits unique advantages in mitigating sarcopenia, especially by modulating these pathways and restoring energy balance (Ali et al., 2022; Zhou et al., 2023; Wang D. et al., 2024; Zhao et al., 2022; Qi et al., 2024). For instance, *A. mongholicus* Bunge [Fabaceae] (Huangqi), a widely used herbal remedy for boosting Qi and blood, is effective in elderly health management (Yao et al., 2024). Studies have demonstrated that Huangqi enhances the phosphorylation of the PI3K/Akt/mTOR signaling pathway, thereby activating muscle protein synthesis and significantly increasing the diameter and thickness of C2C12 myotubes. This provides potential therapeutic benefits for preventing and alleviating sarcopenia in older adults (Yeh et al., 2022). *Codonopsis pilosula* (Franch.) Nannf. (Dangshen), known for its traditional role in tonifying Qi and nourishing Yin, promotes muscle protein synthesis through the activation of the PI3K/Akt/mTORC1 pathway and inhibits the expression of muscle-specific E3 ubiquitin ligases such as MuRF1 and atrogin-1. Moreover, it enhances mitochondrial biogenesis via Sirtuin 1 (SIRT1)/PGC-1α pathway, further improving muscle function (Han and Choung, 2022; Kim et al., 2022). Puerarin, a natural bioactive compound extracted from *Radix Puerariae* (Gegen), activates the Akt/mTOR signaling pathway and inhibits autophagy-related pathways (e.g., LC3/p62), effectively reducing the expression of muscle atrophy markers MuRF1 and atrogin-1 in diabetic rat models. Consequently, it alleviates skeletal muscle atrophy and restores muscle strength (Yin et al., 2021). Similarly, herbal agents such as *Rhizoma Dioscoreae* (Shanyao), quercetin, and black ginseng extract demonstrate muscle-promoting effects by activating the Akt/mTOR axis, facilitating muscle synthesis and inhibiting muscle degradation (Lee et al., 2018; Kim et al., 2023; She et al., 2023).

### 4.2 Mitochondrial metabolism and oxidative stress

In skeletal muscle, mitochondria are the primary source of ROS. Excessive accumulation of ROS triggers oxidative stress, leading to cellular damage, particularly mitochondrial dysfunction, which impairs muscle health and function. Thus, maintaining ROS homeostasis is crucial for preserving mitochondrial integrity (Chen X. et al., 2023). Recent studies have shown that natural monomers, such as naringin (Zhang et al., 2020), Saikosaponin A (Huang M. et al., 2023) can mitigate age-related loss of muscle mass and strength by protecting mitochondria from oxidative damage (Zhang et al., 2020).Further mechanistic insights reveal that myricanol, a natural compound isolated from Myrica species, exerts protective effects on skeletal muscle by activating peroxiredoxin-5 (PRDX5), which reduces ROS accumulation and mitochondrial DNA damage in C2C12 myotubes (Shen et al., 2024).

Mitochondrial energy metabolism dysfunction is a key mechanism underlying sarcopenia, and SIRT1 plays a crucial regulatory role in skeletal muscle metabolism. SIRT1 directly deacetylates PGC-1α and FoxO transcription factors, modulating their nuclear translocation and activity. This regulation promotes mitochondrial biogenesis, maintains muscle mass, and prevents muscle function loss (Alamdari et al., 2012; Gellhaus et al., 2023). Numerous TCM compounds have been identified as SIRT1 activators with anti-aging and anti-sarcopenic effects. For instance, myricanol, a natural compound derived from Myrica species, enhances SIRT1 activity (Shen et al., 2019). Additionally, *Fructus Lycii* (gouqi) has been shown to activate SIRT1, supporting its role in combating sarcopenia and promoting muscle health (Zhou et al., 2024).

In addition to enhancing mitochondrial biogenesis through SIRT1 pathway, natural plant-derived compounds improve skeletal muscle function via complementary mechanisms. Flavonoids extracted from mulberry (*Morus alba L*.) leaves increase the phosphorylation of AMPK, PGC-1α. This activation promotes mitochondrial biogenesis and enhances mitochondrial function. Concurrently, these flavonoids upregulate glucose transporter type 4 (GLUT4) expression on the skeletal muscle cell membrane (m-GLUT4) and in total cellular content (T-GLUT4), improving glucose uptake and insulin sensitivity (Meng et al., 2020). Similarly, berberine and Korean cultivated wild ginseng pharmacopuncture extracts (KCWGP) regulate the expression of mitochondrial-related factors, including PGC-1α, nuclear respiratory factor 1 (NRF1), mitochondrial transcription factor A (TFAM), and SIRT1, through the AMPK signaling pathway. These mechanisms contribute to mitigating pathological changes associated with muscle degenerative diseases (Yu et al., 2018; Hwang et al., 2022).

### 4.3 Inflammation

Chronic low-grade inflammation is another prominent feature of sarcopenia, particularly common among older adults. The Janus kinase/signal transducer and activator of transcription (JAK/STAT) pathway plays a crucial role in immune and inflammatory responses. Its hyperactivation upregulates pro-inflammatory cytokines such as IL-6 and TNF-α, promoting muscle protein degradation. Similarly, NF-κB pathway contributes significantly to inflammation. Excessive activation of this pathway stimulates the expression of muscle atrophy-related genes, such as MuRF-1 and muscle-specific ubiquitin ligase Atrogin-1, further accelerating protein breakdown (Liang et al., 2022).

Magnesium lithospermate B (MLB), a major hydrophilic component of *S. miltiorrhiza* Bunge (Danshen), has shown significant anti-inflammatory effects in animal studies. MLB markedly reduced the levels of TNF-α, IL-6, and their receptor TNFRI in mice fed a high-fat diet and inhibited the activation of the NF-κB pathway, thereby mitigating inflammation-induced muscle degradation (Cheng et al., 2021). Additionally, tanshinone IIA, another active compound in *S. miltiorrhiza* Bunge (Danshen), effectively suppressed lipopolysaccharide (LPS)-induced excessive mitophagy and reduced mtROS accumulation. This helped alleviate inflammation-induced muscle protein degradation and muscle fiber atrophy (Han et al., 2024). Furthermore, the Huanglian Wendan decoction was found to attenuate skeletal muscle damage caused by inflammation through modulating NLRP3 inflammasome pathway (Dong et al., 2021).

### 4.4 Other pathways

The Wnt/β-catenin signaling pathway plays a critical role in muscle generation and repair. Wnt2 promotes the accumulation ofβ-catenin, thereby activating the proliferation and differentiation of muscle progenitor cells such as satellite cells, which supports the repair and regeneration of muscle tissue. Activation of the Wnt/β-catenin pathway also delays cellular senescence and maintains muscle regenerative capacity (Lin et al., 2024).Although studies on TCM targeting the Wnt/β-catenin signaling pathway for treating sarcopenia are relatively limited, existing research highlights promising directions. For instance, polydatin has been shown to promote the proliferation and osteogenic differentiation of human bone marrow mesenchymal stem cells (hBMSCs) via the bone morphogenetic protein 2 (BMP2)-Wnt/β-catenin pathway, indicating its potential in both skeletal and muscular repair (Chen X.-J. et al., 2019). Moreover, Sijunzi decoction may improve bone quality in diabetic mice by modulating the Wnt/β-catenin pathway and maintaining redox homeostasis (Dai et al., 2024).

In East Asia, *P. ginseng* C.A.Mey. (Renshen) has a long history of use, traditionally believed to greatly replenish the body’s vital energy, making it suitable for long-term consumption, with purported benefits such as anti-aging and life extension (Mancuso and Santangelo, 2017). Mountain ginseng (*P. ginseng C.A. Meyer*) is a medicinal plant known for its immunomodulatory properties, anti-muscle damage effects, and promotion of energy metabolism. Studies have shown that mountain ginseng effectively mitigates muscle weight loss and collagen deposition induced by dexamethasone (DEXA) in rats, potentially by inhibiting FOXO3a signaling pathway and downregulating MuRF1 and muscle atrophy atrogin-1 expression (Seok et al., 2021). Another study revealed that long-term intake of Korean Red Ginseng (RGNS) significantly improved aging-related losses in muscle mass and strength. Additionally, RGNS treatment markedly delayed the shift in soleus (SOL) muscle fiber type, slowing the conversion from fast-twitch muscle fibers to slow-twitch fibers (Cho et al., 2022).

Astragaloside IV (ASIV) is the main active component of *A. mongholicus* Bunge [Fabaceae](Huangqi). Research indicates that ASIV can reduce muscle mass loss, muscle fiber cross-sectional area shrinkage, and atrophy in C2C12 myotubes induced by sepsis in mice. The mechanism of action may involve inhibiting the transforming growth factor-beta 1 (TGF-β1)/Smad signaling pathway, thereby reducing the expression of E3 ubiquitin ligases such as MuRF1 and atrogin-1, effectively slowing down muscle atrophy progression (Dai et al., 2023).

Osteocalcin, secreted by osteoblasts, regulates skeletal muscle function through various mechanisms. Its deficiency leads to reduced muscle mass and motor ability in mice. Ginkgo biloba extract can promote muscle regeneration by upregulating osteocalcin expression, providing potential protective effects (Wang B. Y.-H. et al., 2023). Naringin enhances skeletal muscle function by activating the Sp1-estrogen-related receptor gamma (ERRγ) transcriptional axis, promoting an increase in oxidative muscle fibers and improving aerobic metabolism (Lv et al., 2023).

Schisandra chinensis extract also reduces muscle cell apoptosis by inhibiting the expression of caspase-3 and poly (ADP-ribose) polymerase (PARP), enhancing muscle adaptability and slowing the progression of sarcopenia (Kim et al., 2018). These herbal components, through regulating multiple signaling pathways, particularly in slowing muscle degeneration and promoting muscle repair, show immense potential and provide new approaches and therapeutic directions for improving sarcopenia in the elderly.

## 5 Potential side effects and contraindications of traditional Chinese medicine treatments

TCM, including herbal medicine, acupuncture, and qigong, has been widely used for managing sarcopenia and related disorders. Although generally considered safe when administered by qualified practitioners, potential side effects and contraindications should not be overlooked. Herbal medicines can occasionally cause gastrointestinal disturbances, allergic reactions, hepatotoxicity or, nephrotoxicity particularly when used inappropriately or combined with other medications without professional supervision (Yang et al., 2018; Li X. et al., 2022). For instance, certain herbs containing aristolochic acid are nephrotoxic and strictly contraindicated (Ji et al., 2021). Acupuncture, while minimally invasive, may lead to minor adverse events such as bruising, dizziness, or, rarely, infection at needle sites (Huang et al., 2024). Caution is advised in patients with bleeding disorders or those receiving anticoagulant therapy. Qigong and other exercise-based interventions are generally safe but may not be suitable for individuals with severe cardiovascular or musculoskeletal conditions without medical clearance. Furthermore, patient-specific factors, such as age, comorbidities, and concurrent use of Western medications, should be considered to avoid herb-drug interactions and ensure optimal treatment outcomes. Future clinical research should include systematic safety assessments to better inform clinical guidelines and enhance patient safety.

## 6 Integration of traditional Chinese medicine with conventional therapies

Sarcopenia involves reductions in muscle mass, strength, and physical performance, with complex etiologies that include aging-related mitochondrial dysfunction, chronic inflammation, neuromuscular dysregulation, as well as contributions from malnutrition, physical inactivity, and various chronic diseases. Modern medical treatments primarily rely on exercise interventions (such as resistance and balance training), nutritional support (including protein and vitamin supplementation), and, to a lesser extent, pharmacological therapies (such as selective androgen receptor modulators and muscle growth factors). Although exercise and nutritional interventions are currently the cornerstone of sarcopenia management, many elderly patients suffer from comorbid chronic conditions, limited mobility, malabsorption, or poor tolerance to drug therapies. These factors result in limited efficacy of monotherapy approaches, significant individual variability, poor compliance, and suboptimal long-term outcomes.

TCM emphasizes a holistic approach and syndrome differentiation, aiming to reinforce vital energy, activate blood circulation, unblock meridians, and strengthen the spleen and kidneys. TCM treatments not only alleviate symptoms of sarcopenia but also regulate the internal environment and address the fundamental pathological states of the disease. Modern research has confirmed that herbal formulations, acupuncture, Tai Chi, and Baduanjin modulate multiple molecular pathways, including mTOR, PI3K/Akt, and AMPK signaling, promote protein synthesis, reduce muscle inflammation, improve mitochondrial function, regulate gut microbiota, and enhance neuromuscular connectivity. These interventions offer multi-targeted and multi-level therapeutic advantages.

Integrated approaches that combine standardized modern medical management with the individualized treatment principles of TCM allow for comprehensive and flexible intervention strategies tailored to different stages of sarcopenia and patient heterogeneity. While modern medicine provides evidence-based foundational treatments, TCM complements these by improving systemic regulation, delaying the aging process, and enhancing patient compliance and tolerability. Clinical studies have shown that integrative interventions significantly improve muscle mass, strength, and physical function, enhance mobility and activities of daily living, reduce the risk of falls and fractures, and may mitigate the long-term adverse outcomes of sarcopenia. Moreover, TCM therapies are generally well-tolerated with few side effects, making them particularly suitable for long-term management in elderly patients with complex health profiles.

As the aging population increases, single-modality medical approaches are insufficient to meet the diverse needs of sarcopenia management. Integrative medicine, combining TCM and Western therapies, represents a multidisciplinary and comprehensive strategy that not only holds significant practical value but also offers promising directions for future precision medicine and individualized healthcare management.

## 7 Discussion

This review summarized recent advances in the use of TCM for the treatment of sarcopenia, encompassing interventions such as herbal formulas, acupuncture, and traditional exercises like Tai Chi and Baduanjin. These therapies have demonstrated potential to improve muscle mass, strength, and physical performance through a multi-target and multi-pathway approach.

Mechanistically, TCM has been shown to regulate key molecular pathways—including PI3K/Akt/mTOR, AMPK, and NLRP3—involved in inflammation, oxidative stress, mitochondrial dysfunction, and energy metabolism. Additionally, TCM may modulate gut microbiota and enhance neuromuscular connectivity, offering systemic physiological support essential for managing sarcopenia. Acupuncture and traditional physical therapies also contribute by improving balance and neuromuscular coordination, which are vital for fall prevention. Despite promising evidence, most mechanistic studies focus on isolated compounds rather than holistic formulas, and the lack of integrated omics analyses limits our understanding of synergistic effects. Future research should incorporate transcriptomics, metabolomics, and proteomics to elucidate the dynamic network mechanisms of multi-component interventions.

Clinically, several studies have demonstrated that classical formulas such as Buzhong Yiqi Decoction and Sijunzi Decoction, when combined with physical training, significantly improve grip strength, skeletal muscle mass, and gait speed in elderly sarcopenia patients. Some trials also suggest reduced risk of falls and fractures. Compared with monotherapies, integrative approaches combining TCM and Western medicine offer greater adaptability, systemic regulation, and patient tolerance, making them particularly suitable for older adults with complex comorbidities or intolerance to conventional therapies.

However, several challenges hinder the broader application of TCM in clinical sarcopenia management. First, the diagnostic criteria for TCM syndromes remain inconsistent, and classification of common patterns (e.g., Qi deficiency, spleen-kidney Yang deficiency) varies across studies, undermining reproducibility and generalizability. Second, the complex composition of herbal medicines poses uncertainties regarding efficacy and safety. Variation in raw material sourcing, manufacturing techniques, and storage conditions may result in batch-to-batch inconsistency, affecting therapeutic outcomes. Furthermore, many herbal formulas lack standardized dosage guidelines and robust toxicological assessments. In elderly patients with reduced hepatic or renal function, dosage control is especially critical to avoid accumulation-related toxicity.

Moreover, polypharmacy is common in geriatric patients, and co-administration of TCM and Western drugs without rigorous evaluation may lead to herb-drug interactions (e.g., CYP450 enzyme modulation), increasing the risk of adverse effects or therapeutic antagonism. Therefore, future efforts should focus on establishing traceable quality control systems, standardizing dosages, enhancing safety monitoring, and studying pharmacokinetic interactions to ensure clinical efficacy and safety.

The nursing aspect of TCM in sarcopenia care also warrants attention. Incorporating TCM modalities—such as internal herbal administration, topical applications, acupressure, moxibustion, and traditional exercise—into nursing protocols may promote more individualized rehabilitation. Nurses play a pivotal role in patient education, treatment implementation, adherence monitoring, and functional assessments using tools such as the SARC-F or SPPB. Development of standardized nursing pathways that integrate TCM techniques could also be valuable for community-based screening and early intervention.

In summary, TCM provides a multi-dimensional, individualized, and system-oriented approach to sarcopenia management, which effectively complements conventional Western therapies. Future research should prioritize syndrome classification standardization, network pharmacology and omics-based mechanism exploration, multicenter randomized controlled trials, long-term follow-up studies, and integration into clinical nursing practice. With the continuous accumulation of high-quality evidence, TCM is well positioned to be incorporated into international sarcopenia management guidelines, thereby expanding therapeutic options and supporting personalized geriatric care.

## 8 Conclusion

TCM offers a promising complementary approach for managing sarcopenia through multi-target interventions that improve muscle mass, strength, and physical function. Integrating TCM with conventional therapies can address the complex and multifactorial nature of sarcopenia, particularly in elderly patients with comorbidities. Despite encouraging evidence, challenges such as the standardization of TCM diagnostic criteria, limited large-scale clinical trials, and incomplete understanding of multi-target mechanisms remain. Future research should focus on rigorous clinical studies, molecular mechanism exploration, and the development of personalized integrative treatment strategies to enhance clinical outcomes and promote healthy aging.
