# Supplementary material for: Exploring the role of traditional Chinese medicine in sarcopenia: mechanisms and therapeutic advances
Source: Front Pharmacol. 2025 Jun 30;16:1541373. doi: 10.3389/fphar.2025.1541373 (PMC12256475; doi:10.3389/fphar.2025.1541373)
Supplement: Supplementary file 1 [file Table1.docx]

Supplementary materials

Exploring the Role of Traditional Chinese Medicine in Sarcopenia: Mechanisms and Therapeutic Advances

Sheng Xia^2*^, Jianjun Yao^1*^

^1^ Department of Geriatrics, Zhejiang Medical & Health Group Quzhou Hospital(Zhejiang Quhua Hospital), Quzhou 324004, Zhejiang.China.

^2^ Department of Geriatrics, Wenzhou Geriatric Hospital, Wenzhou 325099, Zhejiang, China

**^*^Correspondence:** [yaojianjundoc@163.com,](mailto:yaojianjundoc@163.com,) sxvip2000@yeah.net

# Literature Search Methods

Databases and Time Frame:
To comprehensively review the research progress of Traditional Chinese Medicine in the treatment of sarcopenia, we searched PubMed, EMBASE, Web of Science, Cochrane Library, CNKI (China National Knowledge Infrastructure), Wanfang Data, and VIP Database. The search covered all publications from the inception of each database up to December 2024. Publications were limited to English and Chinese.

Search Strategy:
The search terms included: "sarcopenia," "muscle loss," "Traditional Chinese Medicine," "Chinese herbal medicine," "acupuncture," "qigong," "clinical research," "mechanisms," "Tai chi," and "Baduanjin." These keywords were combined using Boolean operators ("AND," "OR") to ensure comprehensive coverage of relevant literature.

## Inclusion Criteria:

Basic or clinical studies investigating the use of TCM (including single herbs, compound formulas, acupuncture, qigong, etc.) for the prevention or treatment of sarcopenia;

Studies exploring the pathogenesis of sarcopenia, particularly those incorporating TCM theoretical interpretations or related mechanisms;

Various types of publications, including original research, reviews, mechanistic studies, and authoritative guidelines.
